# Breast cancer bone metastases: an orthopedic emergency

**DOI:** 10.1007/s10195-013-0283-6

**Published:** 2013-12-29

**Authors:** Andrea Piccioli

**Affiliations:** Orthopedics Oncology, “Palazzo Baleani”, Azienda Policlinico Umberto I, 00161 Rome, Italy

The aim of the current letter is to discuss the emerging issue of breast cancer (BC) metastatic to bone. Bone is the first metastatic site in >50 % of patients affected by BC. Symptomatic bone metastases (BMs) account for 20–40 % of initially diagnosed metastatic BC, and 70 % of recurrences or previous bone metastatic BCs develop further skeletal lesions [1, 2]. Despite the high incidence of BC, advanced systemic therapies have improved patient’s survival, thus increasing new diagnoses of BMs. Spine and proximal femur are the most common locations of secondary lesions [3]. BMs are the major cause of morbidity in BC patients, being in 75 % of cases responsible for intractable pain and skeletal related events (SREs), such as pathological fractures (in 4–7 % of patients), spinal cord compression, hypercalcemia, anemia, and may be considered as a new emergency with high social and healthcare costs. Peculiar to BC metastatic to bone, is that patients usually show longer life expectancy after occurrence of BMs; moreover, bisphosphonates have significantly changed the natural history of BMs by reducing SREs, so the majority of patients now live with BMs for several years (5-year survival rate >20 % with multiple BMs, 40 % if solitary lesion) [1–3]. An interdisciplinary, patient-customized treatment protocol is widely accepted and performed in dedicated multi-professional care centers called “Breast Units” that aim not simply to provide palliative care, but to guarantee a long survival until complete remission in selected cases. However, once metastatic disease develops, medical treatment resistance is commonly seen and radiotherapy alone does not prevent fractures or neurologic complications in numerous cases, although fractures due to breast BMs respond more favorably to surgical treatment than do fractures secondary to other primary tumors [2]. Thus, BC–BMs represent a major orthopedic issue and multidisciplinary management needs the participation of the orthopedic surgeon as well as the oncologist and radiotherapist. Treatment options and surgical strategies for BMs in general are widely assessed in several reports and guidelines [4, 5]. The aim of orthopedic treatment is to achieve improvement of the patient’s survival and quality of life through pain reduction and early restoration of function. In addition to medical therapies and adjuvant radiation, the role of orthopedic intervention is essential, especially for BMs from BC, and theoretically valuable for every clinical presentation. Patients affected by breast cancer with poor life expectancy (<3 months), without SREs or with a pathological fracture of a non-weightbearing bone, may be initially treated non-operatively (bisphosphonates, analgesics, use of collars, corsets). Absolute indications for surgery are impending or complete fractures of long bones and pelvic girdle, or untreatable pain, instability and spinal compression [4]. Surgical options vary greatly depending on several prognostic factors (Table 1). Operative options for long-bone fractures include intramedullary nailing, plate-screw fixation, conventional or mega-prosthetic reconstruction, eventually in combination with bone cement and other local adjuvants. With spine involvement, decompression alone or additional instrumentation and partial vertebral resection can be performed [3–5]. Minimally invasive and percutaneous treatments with few side effects and complications, such as radiofrequency ablation, cementoplasty, cryoplasty, electrochemotherapy, and MRI-guided focused ultrasound surgery are indicated for patients with no risk of fracture and poor prognosis, while in the case of a solitary lesion and positive prognostic factors, surgery should be more aggressive and a wide total tumor resection should be performed.

**Table 1:** Prognostic factors for BC metastatic to bone

Dimension, histotype, grading of primary tumor
Disease-free interval
First metastasis organs
Estrogen and progesterone receptor status, Her2
Resistance to systemic therapies
Solitary or multiple skeletal lesions
Type of lesions (mixed, lytic, sclerotic)
Skeletal localization
Asymptomatic or symptomatic bone disease (SREs)
Risk of pathological fracture (impending or complete)
Radiation sensitivity
Concomitant non-skeletal metastases
ECOG performance status (Karnofsky score)
Estimated life expectancy above or below 6 months

In conclusion, the epidemiological and clinical impact of BC is remarkable, and it is clear in the scientific community that BC–BM is a unique model for studying bone metastasis. In this context, dedicated orthopedic evidence-based recommendations exclusively for BC–BMs is required.
